# Combination Therapy With Rituximab and Low-Dose Cyclophosphamide and Prednisone in Membranous Nephropathy

**DOI:** 10.1016/j.ekir.2024.08.033

**Published:** 2024-09-12

**Authors:** Coralien H. Vink, Jack F.M. Wetzels, Anne-Els van de Logt

**Affiliations:** 1 Department of Nephrology, Radboud institute of Health Sciences, Radboud University Medical Centre, Nijmegen, The Netherlands

**Keywords:** anti-PLA2R antibodies, immunosuppression, membranous nephropathy

## Abstract

**Introduction:**

Standard treatment with cyclophosphamide (CP) or rituximab (RTX) is suboptimal. We adapted and used the low-dose regimen used in vasculitis (RTX 2 × 1000 mg, CP 1.5 mg/kg/d × 8 weeks, and prednisone [i.v. 2 × 1 g + 3 weeks oral starting at 1 mg/kg]).

**Methods:**

High-risk, anti-PLA2R antibodies (PLA2Rab)-positive patients with membranous nephropathy (MN) were included in this single-arm prospective cohort study. PLA2Rab levels were regularly measured. We report the PLA2Rab kinetics and overall immunological and clinical remission (CR) rate.

**Results:**

We analyzed 26 patients (15 males, aged 57 ± 14 years, PLA2Rab titer 176 [115–460] RU/ml, serum creatinine 128 [102–136] μmol/l, serum albumin 18 [14–21] g/l, and urinary protein-to-creatinine ratio [uPCR] 7.1 [5.7–10] g/10 mmol). Within 8 weeks immunological remission (IR) (enzyme-linked immunosorbent assay < 14 RU/ml) was 88 %. Proteinuria remission after initial therapy developed in 21 patients. Seven patients received renewed therapy, which resulted in proteinuria remission in all. IR and CR were associated with baseline PLA2Rab tertile. Five of 7 patients in need of additional therapy were identified at 4 weeks after start of therapy by PLA2Rab half-life (T_1/2_) > 7 days. Serious adverse events occurred in 4 patients. Adverse events were mild; leukopenia was most frequent.

**Conclusion:**

Low-dose triple therapy induced a rapid IR and CR in most patients. Patients with insufficient clinical response were characterized by high baseline PLA2Rab levels and longer PLA2Rab T_1/2_. Assessment of PLA2Rab levels within 2 to 4 weeks after start of therapy may enable to identify patients who need more intensive therapy.

## Graphical abstract

**Figure:**
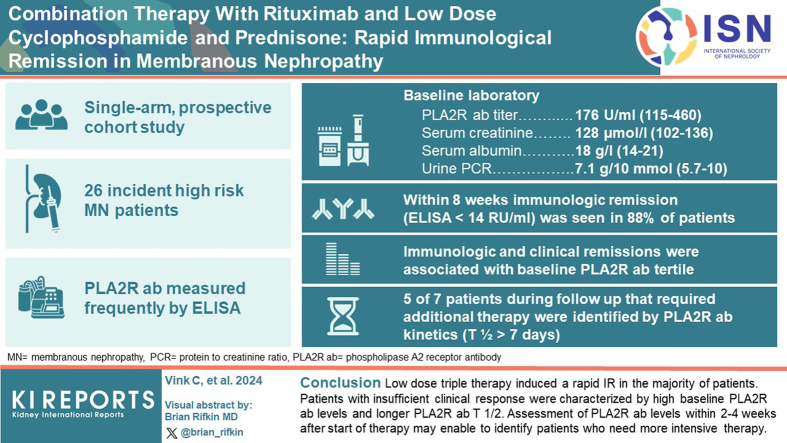

Current treatment modalities in MN are suboptimal: standard dose CP and prednisone (6–12 months) is effective, but associated with toxicity^1^; RTX 1 to 4 g i.v. is considered safe, but associated with a high primary failure rate^2^^,^^3^; and treatment with calcineurin inhibitors (12 months) is associated with high relapse rate.^3^ In a recent study, combination therapy with RTX (cumulative dose of 8 g over a period of 2 years), oral CP (8 weeks), and prednisone (24 weeks) revealed very high CR rates.^4^ Because many patients develop remission with less intensive therapy, a more tailor-made approach is necessary. A regimen consisting of low-dose RTX, CP, and steroids has been successfully used in antineutrophil cytoplasmic autoantibody-associated vasculitis.^5^ In approximately 70% of patients with MN, PLA2Rab are present in the serum.^6^ Measurement of PLA2Rab might allow for more individualized therapy. PLA2Rab levels predicted response to RTX monotherapy.^2^ Moreover, when guiding therapy based on PLA2Rab disappearance, the duration and cumulative dose of CP could be markedly reduced in many patients.^7^ It is evident that IR precedes CR by many months.^8^ Moreover, IR should be the goal of therapy because, except for very rare cases, CR only develops in patients with IR. We have studied the effectivity and tolerability of a low-dose triple therapy regimen in patients with high-risk PLA2Rab-MN. We studied the kinetics of the PLA2Rab response, which may aid in individualization and timely adaptation of therapy in future trials.

## Methods

This was a single-arm prospective cohort study conducted in our academic center. The study protocol was approved by the regional ethical board (CMO region Arnhem-Nijmegen). We adhered to the STROBE criteria for the reporting of cohort studies (Supplementary material). Patients with MN, either initially seen at our outpatient clinic or referred from regional hospitals, were considered for treatment with RTX, CP, and steroids. Suitable candidates, as defined by the inclusion criteria, were adult incident patients (aged > 18 years) with MN and positive PLA2Rab (determined by immunofluorescence tests). Triple therapy was deemed suitable only for patients at high risk for disease progression, using validated biomarkers as described.^9^

We adapted the low-dose regimen used in vasculitis (RTX 2 × 1000 mg, CP 1.5 mg/kg/d × 8 weeks and prednisone i.v. 2 × 1 g + 3 weeks oral [1 week 1 mg/kg, 1 week 0.5 mg/kg, and 1 week 0.25 mg/kg]) for the treatment of patients with PLA2Rab-associated MN in 2020. Eligible patients were informed of the treatment protocol and provided informed consent for therapy, data collection, and analysis. Patients were seen at our center at baseline and 3 months after start of therapy. Interim follow-up visits were done at their local hospitals, using local laboratories for routine parameters (creatinine, albumin, uPCR). Notably, serum samples during these visits were shipped to our center for PLA2Rab assays. Follow-up visits after 3 months were conducted according to local practice. Here, we reports the kinetics of PLA2Rab response and overall immunological and CR rates.

### Definitions and Calculations

PLA2Rab levels were measured using enzyme-linked immunosorbent assay (EUROIMMUN).^10^ IR was defined as PLA2Rab < 14 RU/ml, and strict IR was defined as PLA2Rab < 2 RU/ml. Nephrotic range proteinuria was defined as a uPCR ≥3.0 g/10 mmol. CR was defined as uPCR <3.0 g/10 mmol with a reduction of >50 % from baseline and stable kidney function. Achieving remission includes both partial and complete remission. Relapse was defined as uPCR ≥ 3.0 g/10 mmol after prior CR.

For data analysis, we used descriptive statistics, reporting mean (SD) for parametric data and the median (range) for nonparametric data. Trend tests (Cochran-Armitage Trend test for binary variables, and Jonckheere-Terpstra test for continuous variables) were used to compare baseline and 12-week variables and outcome measures between different PLA2Rab tertiles.

Analyses were performed in R software (version 4.3.1). We used descriptive statistics, reporting mean (SD) for parametric data and median (range) for nonparametric data.

## Results

**Figure 1:**
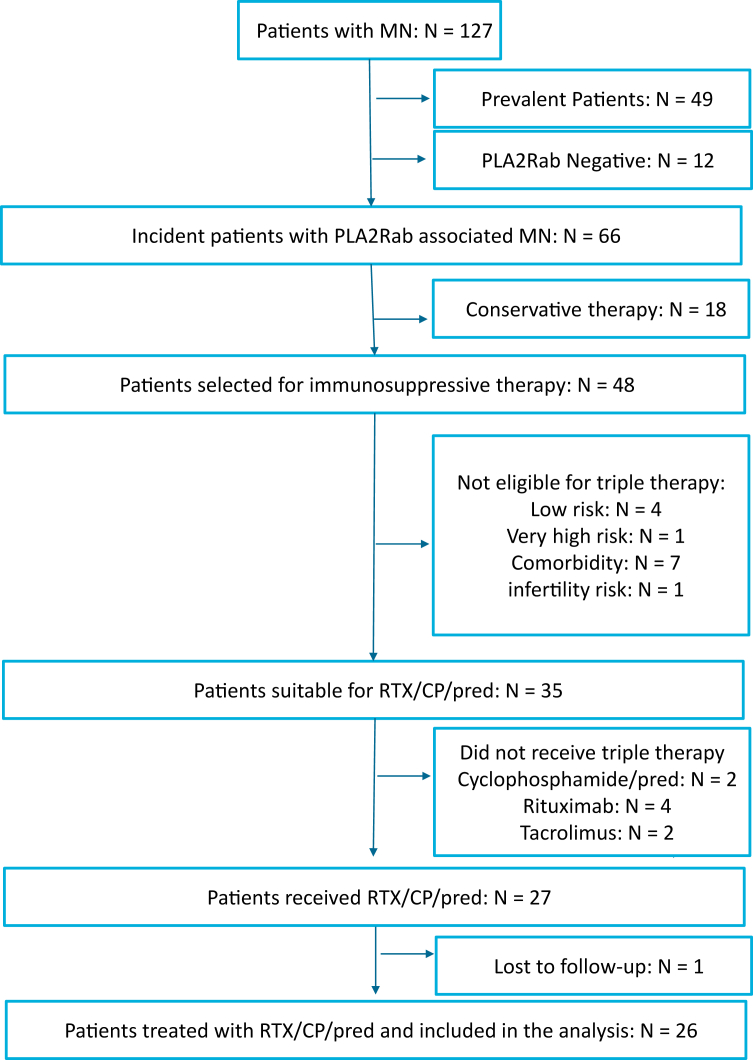
Flowchart of patient inclusion. ∗Some patients were not eligible because of inability to consent (dementia and old age) or comorbidity (malignancy, systemic disease, diabetes). CP, cyclophosphamide; MN, membranous nephropathy; Pred, prednisone; RTX, rituximab.

**Figure 2:**
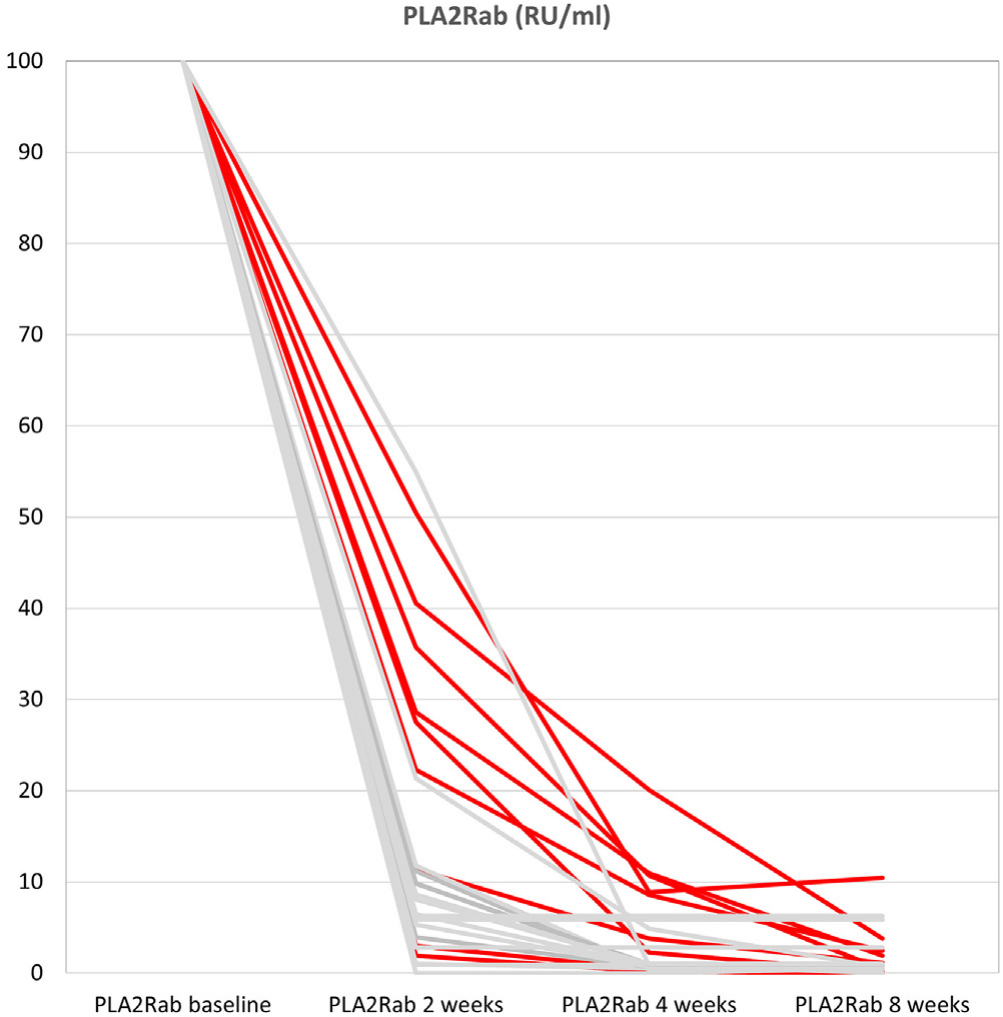
Percentage decrease of PLA2Rab during treatment (baseline titer is plotted as 100%). Patients in the highest tertile of PLA2Rab are depicted in red. PLA2Rab half-life is more than 7 days in 5 of 9 patients in the highest tertile (identifiable by PLA2Rab levels > 25% after 2 weeks or > 6.25% after 4 weeks, respectively).

**Figure 3:**
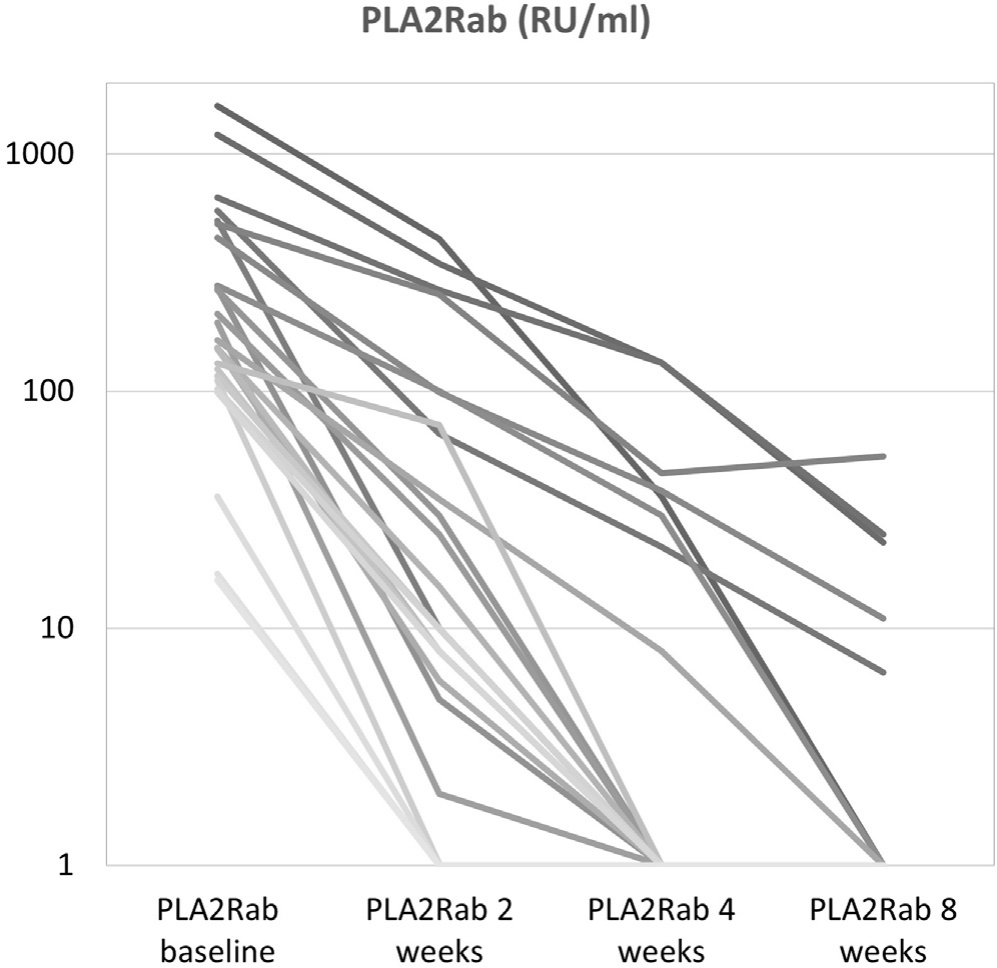
Course of absolute PLA2Rab levels during treatment. PLA2Rab half-life is more than 7 days in 5 of 9 patients with a baseline titer in the highest tertile.

From October 2020 to October 2022, 127 patients with MN were evaluated for immunosuppressive therapy (Figure 1). In 48 incident patients with measurable PLA2Rab, treatment with immunosuppression was advised. Thirty-five patients were eligible for treatment with RTX, CP, and steroids; however, 1 patient refused this therapy, and 7 patients received other immunosuppressive therapies based on physician preference. One patient was lost to follow-up. We analyzed 26 incident patients, of whom 15 were males (58%), with a mean age of 57 ± 14 years. The median PLA2Rab titer was 176 (115–460) RU/ml, serum creatinine was 128 (102–136) μmol/l, serum albumin was 18 (14–21) g/l, and uPCR 7.1 (5.7–10) g/10 mmol. Patients were followed-up for a median of 26 (21–30) months (Table 1). In 3 patients, PLA2Rab levels were imputed at the 8-week timepoints (Supplementary Table S1). We observed a rapid reduction of PLA2Rab levels after the start of therapy (Figures 2 and 3). PLA2Rab levels decreased from 176 (115–460) U/ml to 1 (1–4) U/ml after 8 weeks, representing a Δ% change 98.1 %. After 8 weeks, IR defined as enzyme-linked immunosorbent assay < 14 RU/ml, occurred in 88% of patients, and strict IR defined as enzyme-linked immunosorbent assay < 2 RU/ml, occurred in 73% of patients. During follow-up (and before the start of second-line therapy), proteinuria remission developed in 21 patients (81%). Seven patients received second-line therapy (detailed in Table 2) due to nonresponse (*n* = 5) or relapse (*n* = 2). All patients developed proteinuria remission, which persisted until the end of follow-up in all but 1 patient. PLA2Rab response, the development of IR after 8 weeks, and clinical response were associated with baseline PLA2Rab levels (Table 1). Interestingly, the differences were not merely explained by higher baseline PLA2Rab levels; we also observed a difference in PLA2Rab kinetics, with a T_1/2_ of <7 days in all patients with PLA2Rab levels in the first and second tertiles and > 7 days in 5 of 9 patients in the highest tertile. This difference is illustrated in Figures 2 and 3. Thus, by examining PLA2Rab T_1/2_, 5 of 7 patients who needed renewed therapy were identifiable at 4 weeks after the start of therapy.

**Table 1:** Clinical characteristics and outcome of treated patients, according tertiles of PLA2Rab levels

PLA2Rab tertile	All (*N* = 26)	Low (*n* = 8)	Middle (*n* = 9)	High (*n* = 9)	*P*-value
PLA2Rab (RU/ml)	176 (115–460)	16–124	131–268	273–1600	
Gender (M/F)	15/11	3/5	7/2	5/4	0.24
Age (± yr)	57 ± 14	52 ± 13	62 ± 9	58 ± 18	0.32
sCreatinine (μmol/l)	128 (102–136)	132 (84–177)	128 (115–134)	110 (97–141)	0.48
sAlbumin (g/l)	18 (14–21)	20 (15–25)	17 (14–19)	18 (14–22)	0.48
uPCR (g/10 mmol)	7.1 (5.7–10)	5.8 (4.2–10.0)	7.0 (6.6–8.0)	9.4 (6.1–10.2)	0.90
IR (<14RU/ml) after 8 wk	23	8	9	6	0.01
IR (<2RU/ml) after 8 wk	19	7	9	3	<0.01
PLA2Rab after 12 wks (RU/ml)	1.0 (range 1–65)	1.0 (IQR 1.0–1.0, range 1.0–4.0)	1.0 (IQR 1.0–1.0, range 1.0–1.0)	1.0 (IQR 1.0–10, range 1.0–65)	0.05
Albumin after 12 wk (g/l)	30 (23–32)^a^	31 (23–32)^a^	30 (26–34)	25 (22–30)^a^	0.44
uPCR after 12 wk (g/10 mmol)	4.4 (2.0–6.4)^a^	3.6 (1.5–4.0)^a^	4.2 (1.0–5.9)	6.0 (4.6–8.3)^a^	0.02
PLA2Rab T_1/2_ > 7 d	5	0	0	5	<0.01
FU duration (mo from start immunosuppression)	26 (21–30)	25 (24–31)	28 (20–32)	26 (18–30)	0.70
Partial remission (before additional therapy)	21	8	9	4	
Complete remission (before additional therapy)	10	4	5	1	
Partial remission EFU	25	8	8	9	
Complete remission EFU	9	4	3	2	
Additional therapy^b^	7	0	0	7	<0.01

EFU, end of follow-up; F, female; FU, follow-up; IQR, Interquartile range; IR, immunological remission; M, male; PLA2Rab, anti-PLA2R antibody level; sAlbumin, serum albumin; sCreatinine, serum creatinine; T_1/2_, half-life; uPCR, urinary protein-to-creatinine ratio.

a Albumin and protein-to-creatinine ratio after 12 weeks are missing in 1 patient in the low tertile and 1 patient in the highest tertile, respectively.

b Additional therapy means repeated administration of rituximab (*n* = 5), tacrolimus (*n* = 1), rituximab/cyclophosphamide + prednisolone (*n* = 1) because of persistent nephrotic syndrome (*n* = 5), or early relapse (*n* = 2).

**Tabel 2:** Overview of 7 patients that received additional therapy in the highest tertile

Patient	Baseline			12 wks			2nd line therapy			Rem (mo)
	PLA2Rab	sAlb	uPCR	PLA2Rab	sAlb	uPCR	Interval (mo)	Reason	Type	
1	1600	17	10.1	1	21	4.5	11	Relapse	RTX 2 × 1000 mg i.v.	4
2	1206	27	4.5	12	23	13.3	9	Persisting NS	RTX 2 × 1000 mg i.v.	8
3	658	18	9.7	8	31	8.6	3	Persisting NS	Tacrolimus	3
4	525	11	6.1	1	21	4.1	6	Persisting NS	MPS 3 × 1000 mg iv + RTX 2 × 1000 mg i.v.	4
5	507	19	6.7	65	23	7.6	5	Persisting NS; renal function decline after RTX	RTX 2∗1000 mg i.v.; followed by CP + Pred 2 mo	8
6	444	13	6.1	2	28	4.7	4	Persisting NS	RTX 2 × 1000 mg i.v.	6
7	273	15	11.7	1	27	7.0	14	Relapse	RTX 2 × 1000 mg i.v.	10

CP, cyclophosphamide; MPS, methylprednisolone pulses; NS, nephrotic syndrome; PLA2R, anti-PLA2R titer in RU/ml; Pred, prednisolone; Rem, Remission, interval 2nd line therapy and partial remission; RTX, rituximab; sAlb, albumin in g/l; uPCR, urine protein-to-creatinine ratio in grams/10 mmol.

**Figure 4:**
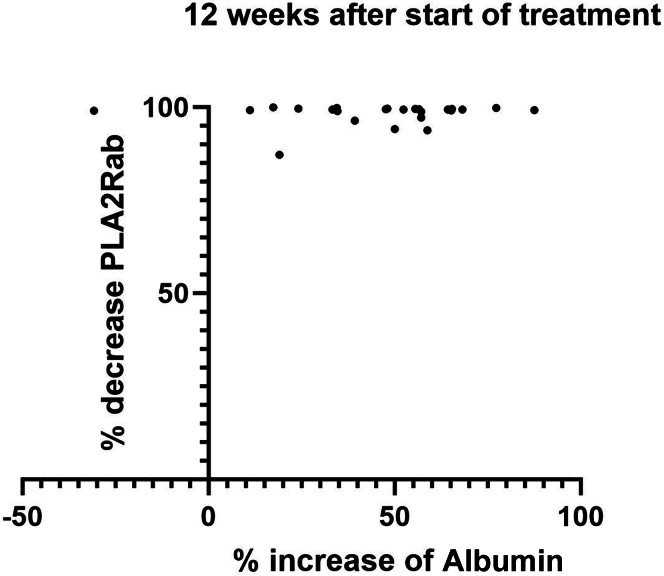
Percentage decrease of PLA2Rab versus percentage increase of serum albumin 12 weeks after start of treatment. Calculation: percentage change = (C12 − C0)/ (Cn − C0), where C12 = concentration at week 12, C0 is concentration at week 0, and Cn = normal concentration (for PLA2Rab = 0 RU/ml; for Salb = 40 g/l).

Information about the course of serum albumin and uPCR after 3 months is provided in Table 1. Although, on average, changes in serum albumin and uPCR were notable at 3 months, further analysis indicated that PLA2Rab response preceded the changes in serum albumin and uPCR (Figure 4). PLA2Rab had decreased by more than 99% in most patients by week 12, whereas changes in serum albumin were quite variable and often less pronounced at this timepoint.

Serious adverse events are detailed in Table 3. The patient who developed a pneumocystis jirovici pneumonia was not prescribed prophylaxis by his treating physician. Another patient experienced a pulmonary embolism due to noncompliance with oral anticoagulation.

**Table 3:** (Serious) adverse events

Serious adverse events	*n* = 1 pneumocystis jerovici pneumonia (2 mo after start) *n* = 1 pneumosepsis (3 mo after start) *n* = 1 Influenza A (2 mo after start) *n* = 1 pulmonary embolism (1 mo after start)
Adverse events	*n* = 6 leukopenia *n* = 1 liver test abnormalities *n* = 1 urinary tract infection *n* = 3 poor sleep quality *n* = 2 loss of hair *n* = 2 nausea *n* = 1 low IgG

## Discussion

Initial therapy combining low-dose RTX, CP, and prednisone resulted in a high rate of IR (88%) and CR (73%). The clinical response rate is notably higher compared to initial RTX treatment (4 g i.v. in MENTOR,^3^ with immunological and clinical response 60%) or low-dose CP (42% clinical response with 8 weeks of CP and steroids^7^). Most striking was the very rapid decrease in PLA2Rab levels; a reduction of >75% was observed in 22 of 26 patients after 2 weeks, and a reduction of >93.5% in 22 of 26 patients after 4 weeks. Extrapolating from these figures, PLA2Rab levels decreased by >50% after 1 week in 21 out of 26 patients (81 %). Although data of PLA2Rab kinetics shortly after starting therapy are limited, existing evidence suggests that triple therapy is superior to RTX monotherapy. For example, Mahmud *et al.*^11^ reported that a 50% decrease in PLA2Rab titers within 1 week occurred in less than 50% of patients treated with RTX. Similarly, the study by Rosenzwajg *et al.* ^12^ found that PLA2Rab levels decreased by more than 50% in only 2 out of 8 patients after 8 days of RTX. In our previous study using CP and steroids, a 50% decrease in PLA2Rab titers within 1 week was observed in <50% (22 of 42) patients.^7^ Despite the theoretical expectation of a rapid initial response with anti-plasma cell therapy, recent research using feltarzamab (anti-CD38) showed that less than 50% of patients had a >50% decrease in PLA2Rab after 7 days.^13^

In this cohort, 27% of patients showed an insufficient clinical response to the low-dose therapy. However, after renewed therapy (primarily with additional RTX pulses), the CR rate improved to 100%, comparable to the high remission rate reported by Zonozi *et al.*^4^ using a higher dose protocol in all patients (cumulative RTX 8 g, and steroids used for 26 weeks) or by Vink *et al.*^7^ using high-dose CP and steroids with treatment duration guided by PLA2Rab disappearance. Identifying nonresponsive patients or those with early relapse early on is crucial, because waiting for 3 to 6 months to observe CR in the presence of persistent nephrotic syndrome can lead to complications and renal injury. All nonresponsive patients were characterized by baseline PLA2Rab levels in the highest tertile. This extends previous studies demonstrating the predictive value of baseline PLA2Rab levels for patients treated with RTX,^2^ where the remission rate was only 30% in the highest tertile. However, using high baseline PLA2Rab levels alone to guide therapy decisions requires further validation, because 2 of 9 patients in this tertile showed a good clinical response. In addition, patients who needed renewed therapy were characterized by insufficient responses after 12 weeks, as indicated by PLA2Rab levels, serum albumin, or uPCR (Table 2). Although our cohort is small and limits firm conclusions, the data suggest that a single biomarker may be insufficient. A model incorporating these biomarkers may prove valuable, although such a model has not been validated for clinical practice.^14^ A model based on biomarkers obtained at 12 weeks after starting therapy might have limited value for very high-risk patients, who would benefit from early identification of nonresponse.

Of note, in this study, treatment was not guided by PLA2Rab levels; all patients received triple therapy, with CP withdrawn after 8 weeks. Renewed therapy was provided only to patients with insufficient clinical response or with relapse. We prospectively collected serum samples and measured PLA2Rab levels to evaluate whether early changes in PLA2Rab levels might determine the effectiveness of the treatment protocol. Our data indeed suggest that measuring PLA2Rab at 1, 2, and 4 weeks after therapy initiation may help identify nonresponders: PLA2Rab T_1/2_ > 7 days identified 5 out of 7 patients who required renewed therapy. Although these findings need validation, they suggest that regular PLA2Rab measurement after starting therapy could be useful in clinical practice for early adjustment of therapy. For patients who achieve CR, regular PLA2Rab monitoring during follow-up may not be necessary. Although an increase in PLA2Rab may precede a clinical relapse, it is not always a reliable indicator, because some patients may have absent PLA2Rab levels at the time of clinical relapse,^15^ or temporary increases not associated with clinical relapse. Further studies are needed, but we advocate for monitoring based on clinical parameters in such patients.

Our treatment regimen was associated with adverse events. Four patients experienced serious adverse events related to immunosuppression and increased infection risk. The overall serious adverse event rate is comparable to that reported in trials using combined CP and prednisone, RTX monotherapy, or calcineurin inhibitors.^1^^,^^16^ Adverse events were mostly related to CP use (eg, leucopenia, nausea, and liver test abnormalities) and were generally manageable. Notably, because our regimen included a relatively low dose of prednisone, we did not observe typical steroid side effects such as diabetes, Cushing syndrome, or skin abnormalities.

Our regimen is open to debate. We used high-dose methylprednisolone pulses (1000 mg), based on the original Ponticelli protocol,^17^ which has been our local practice for over 30 years. However, there is no evidence supporting such high doses, and Spanish researchers have proposed that lower doses of i.v. methylprednisolone pulses (125–250 mg) might be sufficient.^18^ We suggest considering lower doses of i.v. methylprednisolone pulses in our regimen. The use of oral CP is also debatable. In treating vasculitis and lupus nephritis, i.v. CP is often preferred, because it may be as effective as oral CP but with a lower cumulative dose. Indeed, in the CYCLOPS study,^19^ in patients with antineutrophil cytoplasmic autoantibody-associated vasculitis, no difference was found in initial response to therapy. However, more relapses occurred in the i.v. arm, indicating that long-term outcomes might depend on cumulative dose. A randomized trial comparing i.v. CP (750 mg/m^2^ monthly for six months) combined with i.v. methylprednisolone to the standard Ponticelli regimen showed the latter to be superior.^20^ Noncontrolled studies have suggested the efficacy of i.v. CP in combination with oral prednisolone; however, these studies are not strong due to their uncontrolled, retrospective nature and moderate risk profiles. In addition, data on PLA2Rab levels are lacking. An example is the study of Luzardo *et al.*^21^ Luzardo reported outcomes in 55 patients treated with i.v. CP in a dose of 15 mg/kg once in month 2,4, and 6 as part of the cyclical regimen. The retrospective study included 55 incident patients treated in Uruguay in the period 1990 to 2017. Treatment was started early (2.7 months after biopsy) and baseline characteristics were suggesting moderate risk profile. Cumulative incidence of complete and partial remission was 76%, and nonresponder rate of 24%, 5 of whom started renal replacement therapy. Further studies are needed to evaluate the use of i.v. CP in MN treatment and determine the optimal dosing schedule.

Our study focused on evaluating the efficacy of the triple regimen. Considering that we were specifically interested in PLA2Rab kinetics, our study included only patients with measurable PLA2Rab. However, about 30 % of patients with MN lack PLA2Rab.^6^ Although new antibodies have been detected, most lack commercial assays for clinical use. Because PLA2Rab was not used to guide therapy, a similar regimen could be employed for PLA2Rab-negative patients, with decisions on renewed therapy guided by clinical parameters.

Limitations of our study include the small number of patients and lack of a validation cohort. Besides, we have calculated PLA2Rab T_1/2_ assuming linear kinetics, using data obtained at baseline and after 2 and 4 weeks.

In conclusion, a triple therapy regimen combining RTX, and low-dose CP, and prednisone is effective in MN, though a quarter of patients require additional therapy. Detailed monitoring of PLA2Rab kinetics might allow for early prediction of response (after 2–4 weeks), facilitating further individualization of treatment and timely adaptation. We suggest that PLA2Rab-guided therapy should be studied in clinical trials.

## Disclosure

All the authors declared no competing interests.

## Supplementary Material

Supplementary File (PDF and Excel)

**Supplementary Table S1.** Table with clinical and immunological data.

Strobe checklist
